# CellMate—A Deep Learning-Assisted Single-Cell Data Processing Platform

**DOI:** 10.1021/acs.analchem.5c07205

**Published:** 2026-02-13

**Authors:** Felix Friedrich, Cátia Marques, Ingela Lanekoff

**Affiliations:** † Department of Chemistry for Life Sciences, 8097 Uppsala University, Uppsala 75 123, Sweden; ‡ Center of Excellence for the Chemical Mechanisms of Life, Uppsala University, Uppsala 75 123 Sweden

## Abstract

Mass spectrometry-based single-cell metabolomics (SCM) reveals the inherent heterogeneity of individual cells among seemingly identical cell types. Fast-scanning and high-resolving mass analyzers provide the sensitivity and specificity required to probe minuscule amounts of biological material. However, acquiring data from hundreds of individual cells to achieve statistical power results in complex data sets. This challenge is compounded by the limited availability of specialized data analysis tools for single-cell metabolomics, as many techniques depend on the use of specialized sampling and ionization probes. This results in incompatibility with conventional metabolomics data processing tools. Here, we present CellMate, a MATLAB-based data processing platform designed for single-cell metabolomics using direct infusion techniques. CellMate comprises identification and peak alignment of detected metabolites in an intuitive graphical user interface. CellMate supports customizable quantitative, targeted, and nontargeted metabolomic workflows. The untargeted workflow is enabled by a novel deep learning-based image classification algorithm that effectively distinguishes endogenous metabolites from background species. The source code, along with a compiled installer, is available at github.com /LanekoffLab/CellMate. We believe that CellMate represents a significant advancement in the single-cell metabolomics toolbox, enabling comprehensive data extraction of precious metabolite information from single cells.

## Graphical abstract

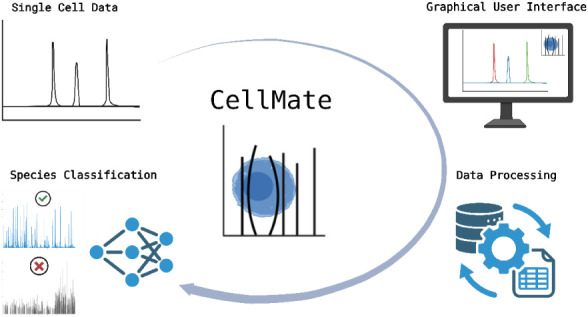

## Introduction

Chemical cell-to-cell heterogeneity is crucial and facilitates adaptation to ensure the survival of a cell population in dynamic environments.^1^ For example, it enables a diverse cellular response to environmental stimuli, which can influence the function, differentiation, and disease progression in biological systems.^2,3^ However, chemical analysis of individual cells is challenged by their small size, picoliter volume, and low amounts of each molecule. Nevertheless, single-cell (SC) analysis has been successful across various omics fields, including transcriptomics,^4^ proteomics,^5^ and metabolomics.^6^ For example, cellular subpopulations within seemingly similar cells have been detected and identified as crucial for understanding drug resistance,^7^ gene expression,^8^ and the dynamics of cellular transitions from healthy to diseased states.^9^ While transcriptomics can be achieved by amplification prior to the detection of four known bases, and proteomics relies on the identification of molecules defined by twenty-letter codes, metabolomics requires more versatile methods. For metabolomics, the methods must account for various molecular structures comprised of similar atoms while exhibiting a wide range of physicochemical properties, which challenges analysis.

Single-cell metabolomics (SCM) requires instrumentation with high sensitivity. This is achievable through the combination of mass spectrometry with innovative and comprehensive sampling and ionization techniques.^6^ Specifically, various ionization methods, including matrix-assisted laser desorption ionization (MALDI), secondary ion mass spectrometry (SIMS), and electrospray ionization (ESI)-based techniques, have all reported detection of small molecules from individual cells.^10,11^ With ESI, sampling is enabled under ambient atmospheric conditions, which is the basis for many specialized sampling probes^12^ and microfluidic-based methods.^13^ For example, using a nanoESI-APCI hybrid ionization source, prostate cancer stem cells were distinguished from nonstem cancer cells, revealing significant metabolic heterogeneity and pathway alterations, including the TCA cycle, amino acid metabolism, and arginine biosynthesis.^14^

Single-cell experiments require sampling of hundreds to thousands of individual cells to achieve statistical significance.^15^ This results in large and complex data sets that require efficient data extraction and handling for subsequent statistical analysis.^16^ While SC proteomics can be performed on established data processing platforms,^17,18^ tailored data processing workflows have been developed for SCM techniques.^19^ Ambient SCM is often based on continuous or pulsed sampling with direct infusion mass spectrometry (DIMS), where individual cells are typically recognized using the detection of ions from known endogenous molecules.^20,21^ However, the lack of chromatographic separation results in complex mass spectra with thousands of peaks, including a significant fraction of background species that originate from the solvent, contaminants, the environment, or ionization artifacts.^22,23^ Thus, efficient data processing methods are required to remove background signals while retaining all *m*/*z* values that originate from endogenous molecules present in the cells. Furthermore, dynamic alignment of *m*/*z* values is required in Fourier transform mass spectrometry (FTMS), where static binning does not adequately match the inverse relationship of resolution and *m*/*z* ratio.^24^

Developed data processing platforms address the cell event detection, *m*/*z* alignment, and background removal using statistical approaches, including detection frequencies and signal-to-noise ratios (S/N).^18,19^ However, statistical methods often involve parameter optimization, leading to a trade-off between analyte coverage and background removal.^19^ For similar problems in chromatography and bioinformatics, machine learning (ML) and deep learning (DL) methods have been adopted to address this shortcoming for peak picking algorithms and peak quality assessment.^25–27^ Besides the construction and training of new networks, existing trained configurations can be fine-tuned or tailored to a new task through transfer learning. This approach adapts a trained model to a different task, enabling model optimization with smaller data sets and shorter training times.^28^

Here, we describe a new analysis platform for single-cell data, CellMate, that can handle continuous, direct-infusion experiments with repetitive events, such as sampling hundreds of cells in a single data file. The user-friendly graphical user interface (GUI) allows automatic processing for targeted and untargeted approaches. An integrated image-classification DL model overcomes the trade-off between analyte coverage and background removal by transfer-learning a pretrained model on categorized extracted ion chronograms. The integrated DL saves valuable analysis time by automatically removing most background species. We anticipate that this platform will be a valuable resource for the growing SCM community utilizing ambient ionization methods.

## Materials and Methods

### Data Collection

Cells were deposited in a 10 × 10 array with the CellenONE (Cellenion, France). After cell deposition, the slides were snap-frozen by placing them on a liquid nitrogen-cooled metal surface and stored in glass vials at −80 °C. Six SCM data sets were acquired in touchdown mode using a tapered PA nano-DESI probe as previously described.^29^ The solvents and ionization modes for data set 1 and 4 were MeOH:H_2_O (9:1) with 0.1% formic acid in positive mode, for data set 2 and 5 were ACN:IPA (9:1) with 0.1% formic acid and 10 ppm monoisotopic silver in positive mode, and for data set 3 and 6 were MeOH:H_2_O (9:1) in negative mode. An IQ-X Tribrid instrument (Thermo Fisher Scientific, USA) was used to acquire data in positive and negative mode between *m*/*z* 75–1000 for the MeOH:H_2_O (9:1) solvent and *m*/*z* 200–2000 for the ACN:IPA (9:1) solvent, at a mass resolution of 240 000 (*m/Δm* at *m*/*z* 200).

### Development

A standard workstation computer with an Intel Core i7–12700 Processor and 32 GB of RAM with an NVMe SSD hard drive was used throughout the development and testing. The program was developed in MATLAB 2023b (MathWorks Inc., USA) with the Deep Network Designer and the App Designer app. The pretrained models, Inception-v3, Inception-ResNet-v2, and EfficientNet-b0, were downloaded into the MATLAB environment. The last *fully connected layer’s* output size was unlocked, meaning the pretrained weights were removed and the size was changed to 3 to enable multiclass classification in the *classification layer*. For training, the mini-batch size was reduced to 64 to fit within the memory of the used PC.

### Model Training

The six different SCM data sets were used to develop and validate the DL model. Prior to development, the *m*/*z* values were manually assigned as either high signal-to-noise (S/N), low S/N, or background. Data sets 1–3 were combined to train the model. In total, these data sets contained 3684 high S/N, 3952 low S/N, and 4044 background images. Within the training data, 30% was reserved for validation during the training process, and training was stopped early if no improvement was observed for five consecutive validation checks (evaluated every 30 iterations). EfficientNet-b0 was used as the final model without additional hyperparameter optimization because it already achieved >95% accuracy, sensitivity, and specificity. Data sets 4–6 were used for testing.

### Results and Discussion

Single-cell metabolomics generates large amounts of complex data that requires robust and precise processing. When using tapered PA nano-DESI MS in touch-down mode for single-cell metabolomics, the probe is parked on a cell, and data is continuously acquired until the metabolome is exhaustively extracted.^29^ Following, the next cell is moved to the sampling probe, and data from the background is detected in the meantime. This generates a cellogram that contains several cellographic peaks, where each peak corresponds to data collected from an individual cell.^30^ Here, a typical cellogram contains 100 cells that are detected with a detection frequency of 3 cells per minute.^29^ A similar data structure would be expected from other direct fusion MS single-cell sampling platforms. Thus, an efficient data handling tool is required for rapid data extraction and selection of endogenous molecules originating from the individual cells.

We have developed CellMate in the MATLAB environment with the goal to efficiently and reproducibly handle this data by 1) identifying the data originating from cell sampling, 2) extracting the *m*/*z* and intensity values from the data of the sampled individual cells, and 3) reliably selecting *m*/*z* values that originate from endogenous metabolites sampled from the cells (Figure 1). The CellMate app combines all necessary processing steps in one place, which enables a streamlined workflow from data import, through extraction and selection, to aligning files from multiple experiments.

**Figure 1:**
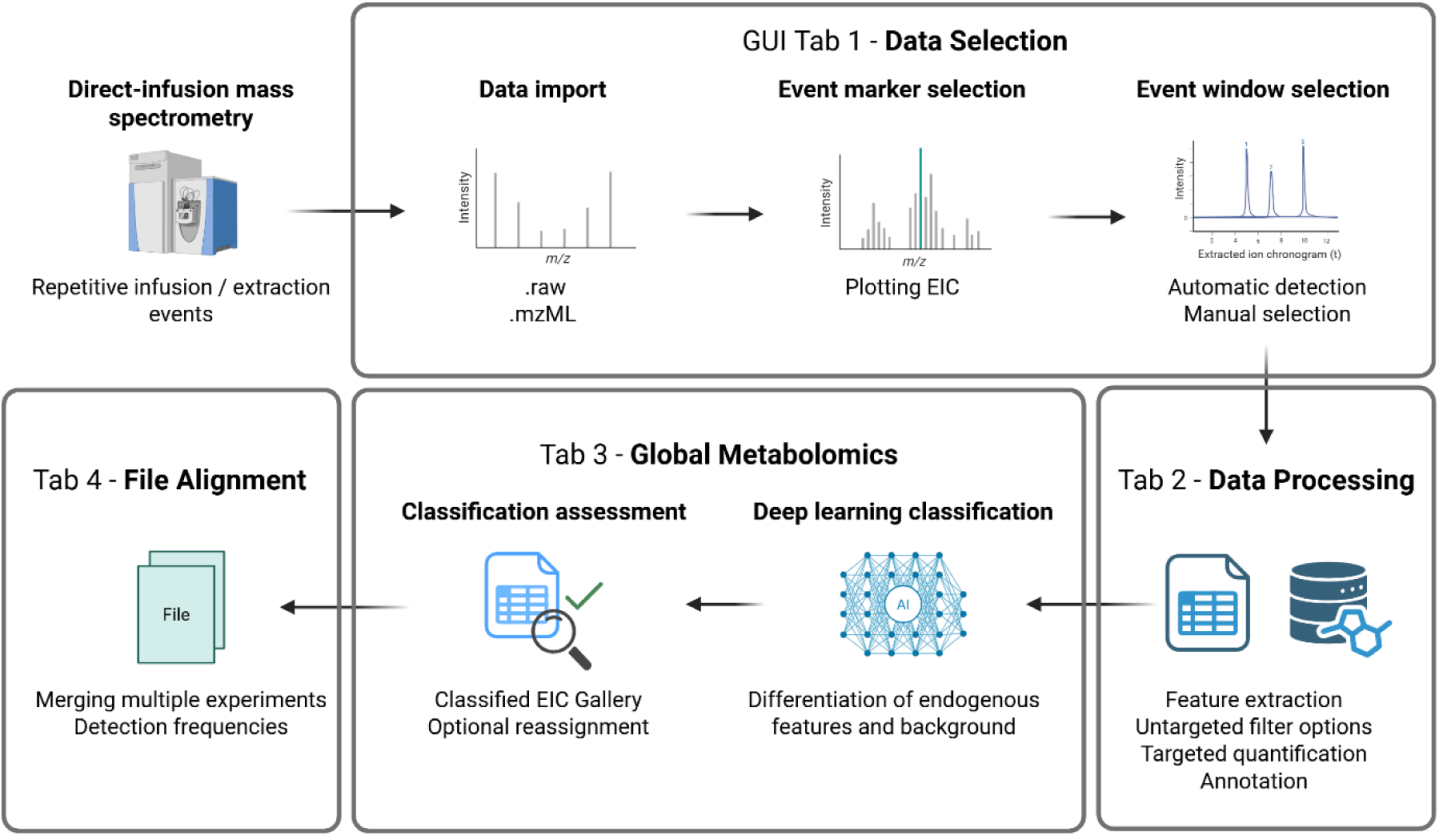
Schematic representation of the streamlined data processing workflow of CellMate. In tab 1 of the GUI, data is imported via either ThermoFisher’s .RAW format or the standard .mzML format. One *m*/*z* of an endogenous species is selected to plot an extracted ion chronogram (EIC) and visualize the single-cell measurements across the entire file as a cellogram. In tab 2, the data is processed with targeted and untargeted approaches, enabling filtering, normalization, quantification, and annotation. In tab 3, the nontargeted and targeted approach can be supported by a DL model that classifies *m*/*z* values into endogenous or background origin. In tab 4, multiple files can be aligned and filtered across the experiments.

### Tab 1—Data Import and Extraction

The first step of the CellMate workflow involves importing data and selecting data extraction windows. The single-cell data easily comprises several gigabytes (GB) and can be imported into CellMate either as .RAW files through the RawFileReader.NET dynamic link libraries (ThermoFisher) or as .mzML files. Most vendor formats are convertible to the .mzML format, making CellMate versatile and vendor independent. In the conversion process to .mzML, the data is centroided and compressed by a factor of 3, which reduces storage size and increases speed compared to noncompressed data.^31^ However, compared to .RAW, instrument-specific information and metadata are lost during the conversion. This includes the noise and baseline level of the respective *m*/*z* values, as well as the frequency/transient domain for in-house Fourier transformation and calibration when using the unreduced .RAW data format. Despite the data compression of .mzML, the different data storage structure provides a reading time for the .RAW format that is eight times faster for a similar size, with linear regression values of 0.006x compared to 0.049x for the .mzML format (Figure S1).^32,33^ Although the differences are negligible for smaller files, importing file sizes exceeding 1 GB is more efficient with the .RAW format, which is therefore the preferred format when possible.

With 100 cellographic peaks in one cellogram, the underlying mass spectra detected from each cell need to be extracted for processing, evaluation, and reporting. The shapes of the cellographic peaks will vary for different *m*/*z* values, due to different extraction and ionization efficiencies and abundances of the endogenous molecules. Therefore, to ensure full coverage, it is ideal to expand the cellographic peak when extracting the data. The first tab of the CellMate GUI allows for the selection of individual cellographic peaks either manually or automatically. The manual selection requires the user to upload a .csv file with start and end times of each extended cellographic peak. For the automated selection, a user-selected *m*/*z* value, corresponding to an endogenous molecule, is used by CellMate to identify peaks in the cellogram based on user-set parameters, including intensity or signal-to-noise (S/N) thresholds and minimum distances as the refractory period between individual cellographic peaks (Figure 2 and Figure S2). In CellMate, the user can also manually add or delete individual peaks (Figure S3). Following peak identification, the sum of the intensities for each *m*/*z* value from each cell is calculated.

**Figure 2:**
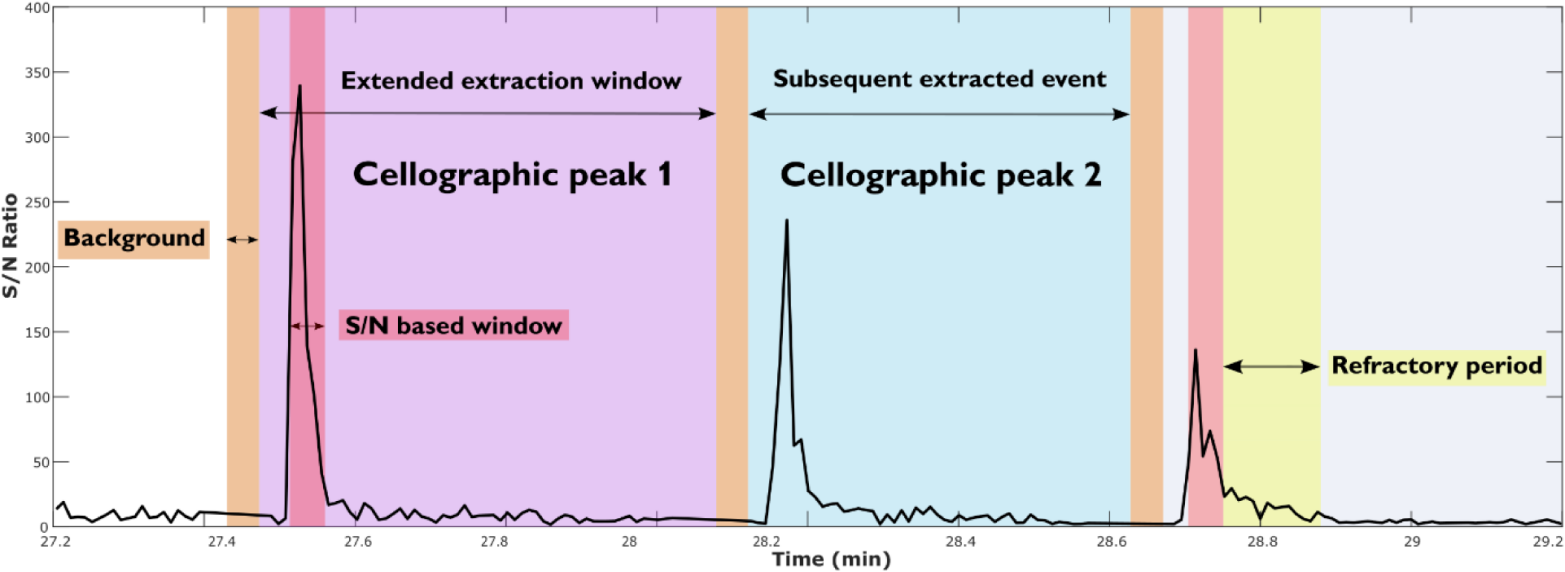
Schematic of automated detection of cellographic peaks corresponding to data from individual cells. Two extended peaks, (violet and blue), are defined based on the S/N increase (red) of the selected endogenous *m*/*z* of the cellogram. Background spectra for each individual cell are taken before the peak (orange), and the set refractory period (yellow) marks the extended peak where no new cell can be registered. The extended cellographic peak encompasses all spectra between the background for the identified peak and the start of the background for the subsequently identified peak. The peaks can be extended further by preceding scans to cover the event time before a sufficient S/N ratio is achieved.

The accuracy and efficiency of the automatic identification function were evaluated using six different SCM data sets with a known number of cells and using optimized parameters for each data set. The optimized S/N values varied from 10 to 40, depending on the relative abundance of the selected cell marker to detect most cellographic peaks. When a too high S/N value was chosen, peaks with low abundance were lost, while a too low S/N value led to the splitting of peaks. The splitting of cellographic peaks could be compensated for by increasing the refractory period. However, if the refractory period becomes too long, subsequent cellographic peaks within that time window are ignored. Thus, the S/N value and refractory period are interdependent. Additionally, it is highly important to select an appropriate cell marker, i.e., a *m*/*z* value corresponding to an endogenous analyte that is highly abundant in the mass spectrum. The selected species are data set and operator-dependent and need to be assessed for each data set. For example, the use of protonated PC 34:1 (*m*/*z* 760.586) (Figure S3), positively charged choline (*m*/*z* 104.107), positively charged oleamide + Ag^107^ (*m*/*z* 388.176), or negatively charged Glu–H-H2O (*m*/*z* 128.035) was successful in the evaluated data sets for the respective experimental conditions. Specifically, the results show that the algorithm successfully identified a minimum of 90% of cells (Data set 1) to 99% (Data set 2) (Table 1). This suggests that the algorithm for automatic cell identification was robust.

**Table 1:** Optimized Parameters and Success Rates of Automatic Identification of Cellographic Peaks in Six Different Datasets^a^

Condition	Cell marker*m*/*z*	Intensity threshold (S/N)	Refractory period (min)	Identified cells	Success rate [%]
Data set 1	760.588	40	0.20	84/93	90
Data set 2	388.176	10	0.10	98/99	99
Data set 3	128.035	40	0.24	84/90	93
Data set 4	760.586	40	0.20	75/79	95
Data set 5	388.176	25	0.15	83/85	98
Data set 6	128.035	27	0.20	48/50	96

a The identification rate is calculated from the ratio of correctly identified cellographic peaks and the known total number of individual cells used in the experiment.

### Tab 2—Data Processing with Alignment and Filtering

Data processing from a cellogram of >1 h and several thousand extracted high-resolution *m*/*z* values is computationally intensive. The processing time is dominated by the alignment of *m*/*z* values for targeted and untargeted approaches, which is necessary because of the intrinsic mass shift of *m*/*z* values between scans. The alignment is performed using our previously developed algorithm in the imaging data analysis platform i2i, which has proven efficient and retains the high-resolution details of FTMS instrument data.^31^ In short, the algorithm groups *m*/*z* features into feature groups within a given ppm tolerance. In CellMate, computational time is reduced by splitting the alignment into two steps: first, by aligning *m*/*z* values within each cellographic peak, and second, by aligning the integrated *m*/*z* values of all cellographic peaks (Figure S4). Throughout the data processing, intensity thresholds and different detection frequencies (DF) filters of *m*/*z* values are applied. After the first alignment, the number of *m*/*z* values is reduced by a fold change (FC) filter that acts as an S/N ratio filter. The FC is calculated by dividing the highest signal intensity within a cellographic peak by the averaged intensity (default ten scans) before the peak inside the background window for each *m*/*z* species. The FC filter can be varied, but is set to 3 by default to reduce noisy *m*/*z* species that are affected strongly by the background signal. By reducing the number of *m*/*z* values included in the alignment process, computational and time demands are decreased to ensure smooth and time-efficient data handling.

In the targeted workflow, a target list with *m*/*z* values is used and can be run at low filter thresholds.^31^ Additionally, internal standards (IS) included during analysis can be easily used in the targeted workflows for quantification (Table S1).^34,35^ However, for nontargeted analysis, the complex spectra, including background ions, inflate the number of *m*/*z* values and computational demands, making the use of optimal filtering settings for data extraction essential. After aligning and reducing the data of each cellographic peak, additional filtering is required to select *m*/*z* values that correspond to monoisotopic endogenous species. In addition to DF and signal-to-noise ratios,^36^ filtering options include exclusion of known solvent blanks, internal standards, or^13^C-isotopologues. Furthermore, mass defect filtering (MDF) excludes specific mass defect ranges, which is broadly applied in mass spectrometry.^37^ For example, McMillan et al. used MDF to filter out solvent peaks, salt clusters, and multiply charged species by comparing experimental data (human plasma) against the human metabolome database (HMDB).^38^

We evaluated the cumulative effect and filter capacity in the nontargeted workflow using data set 1. Specifically, we selected increasing thresholds of the FC and DF filters, starting at 3 and 33%, respectively. Higher FC values require higher S/N ratios of species between the cellographic peak and before, and discard species with a high background signal. Raising the DF selects only species that were integrated during the first alignment and passed the FC filter at a specific rate. Sporadically found species, occurring in less than one-third of the cells, are filtered out by default, although this can be changed when searching for rare metabolites. The optional filters, such as MDF, and the application of exclusion lists for IS or solvent blanks are applied only once. The filters are cumulatively applied in an order, which is assumed to minimize the loss of endogenous features, such as starting with low filter thresholds and applying deisotoping and exclusion lists. The results are presented as the percentage of retained endogenous and background features at consecutive filtering steps and thresholds (Figure 3). Specifically, about 2700 background species and 95 endogenous species were processed in the initial step at a DF of 33%. By gradually applying the different filters cumulatively, the background species are gradually reduced while retaining most of the endogenous species. In that filtering sequence, the first loss of endogenous species, at a rate of 3%, occurred during the MDF. Background species are reduced by about 50% with DF and FC filters at default settings and the optional filters, while preserving almost all endogenous species. This leaves roughly 1400 *m*/*z* species after filtering. To extract 97% of endogenous species, it was found that the data needed to be manually inspected and characterized as background or endogenous metabolites (Figure S5).

**Figure 3:**
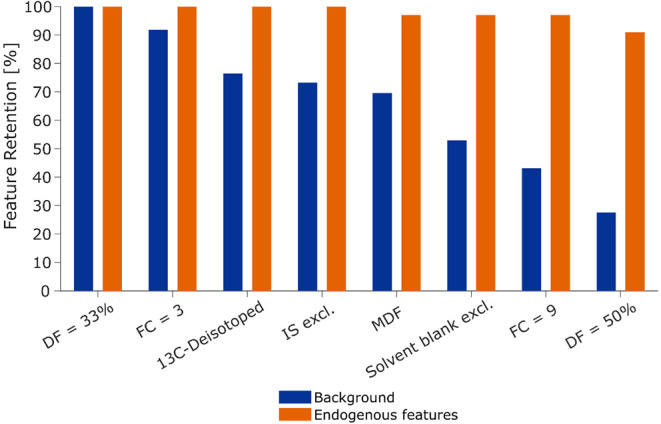
Evaluation of filtering effects on data set 1. The percentage of feature retention for background species (blue) and endogenous features (orange) at different filtering steps for data set 1. The first filter step, with a DF of 33%, serves as a reference for feature retention, encompassing ∼2700 background species and 95 endogenous features. The filters are applied consecutively to reduce the total feature pool while retaining most endogenous features. DF (by default 33%), FC (by default 3), ^13^C-deisotoping, IS exclusion, MDF, and solvent blank exclusion, together reduce background species by about 50% while preserving nearly all (97%) endogenous features at default settings. Higher thresholds further reduce the background to about 30% but reduce endogenous features to 91%.

Increasing the filtering threshold of the detection frequency and fold change can reduce background species, but risks losing endogenous features. In this data set, the background was reduced to about 30% but the retention of endogenous species shrank to 91%. Thus, statistical filtering has an inherent trade-off between coverage (endogenous species retention) and specificity (background removal), requiring adjustments for every data set. Aiming for high coverage of analytes requires low filter thresholds, which can result in several hundred to thousands of extracted *m*/*z* values. Since manual inspection is time-consuming, a more productive approach to extract nearly all endogenous species at default thresholds without excessive manual review and filter adjustment is desirable and explored below.

### Tab 3—Deep Learning-Assisted Nontargeted Data Analysis

As an alternative to using filters to remove background ions and retain endogenous species, the EIC can be assessed globally by examining the profiles of different *m*/*z* values over time. By categorizing the EIC profiles, it is possible to assess if their origin is from a cell or the background. Here, we use three categories: High S/N, low S/N, and background (Figure 4 and Figure S6). The high S/N profile displays a sharp, characteristic profile that is associated with the time of cellographic peaks and minimal background (Figure 4a). In more detail, the sharp peaks are clearly separated from each other due to the very low baseline intensity level, which is several magnitudes lower than during the contact with the single cell. The associated *m*/*z* species show high abundance, as indicated by both the measured intensities and the peak frequency across the experiment. The low S/N displays a more diffuse profile that is still associated with the time of cellographic peaks but has a higher background (Figure 4b). Here, the peaks are less prominent and accompanied by a notably elevated background, potentially failing to achieve even a single order of magnitude of separation from the peak intensities. Moreover, the peaks can be low-abundance, resulting in lower intensities than those of the high-S/N species and a lower peak detection frequency across the experiment. In contrast, the background shows no correlation with the cellographic peaks and displays a wide variety (Figure 4c). Background intensities may remain static or fluctuate significantly across the spectrum. In certain instances, the intensity can spike once or twice during an experiment, mimicking the appearance of cellographic peaks. This creates borderline cases (Figure S6d) that are difficult to classify as either background or endogenous, particularly if a spike coincides with the timing of single-cell contact.

**Figure 4:**
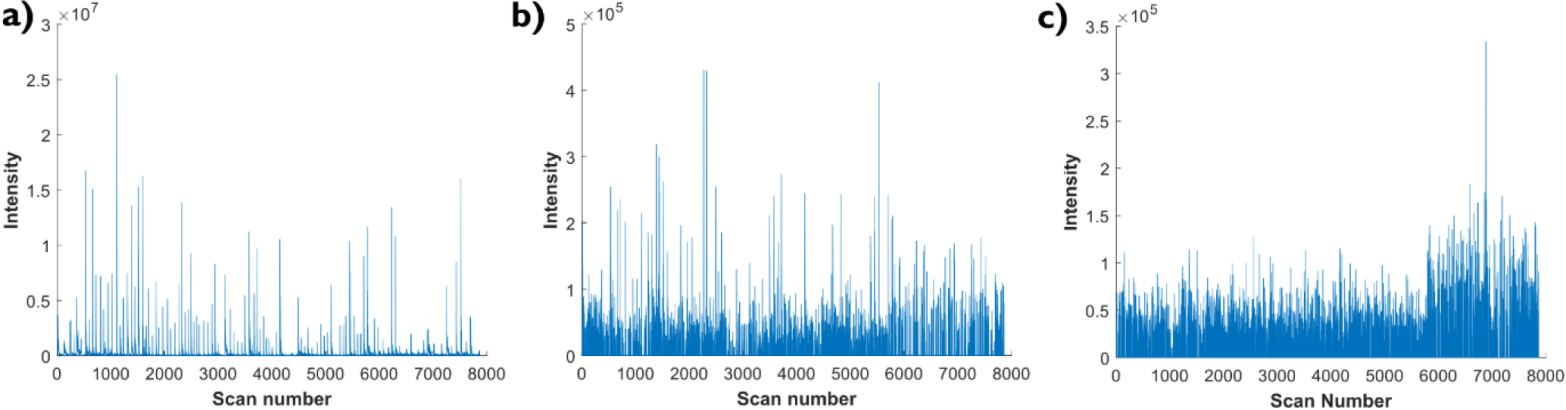
Different EIC profiles for different *m*/*z* values. a) High S/N—endogenous species at *m*/*z* 104.107 (choline^+^), b) Low S/N—endogenous species with significant background at *m*/*z* 118.086 (Valine+H^+^), and c) background—at *m*/*z* 104.058 with no visible correlation pattern to sampled cells.

Based on these characteristic profiles, we developed a deep learning (DL)-based method for classifying the origin of detected *m*/*z* values based on their EIC profiles. To train the DL method, all EIC profiles were manually assessed and sorted into three classes: high S/N, low S/N, and background. Ambiguous categories, such as the low S/N category that includes less definable EIC profiles, are utilized by multiple models when a clear distinction is difficult.^25,26,39^

When the manual classification was finalized, different pretrained DL image classification models were evaluated to avoid developing a DL model from scratch and incurring the computationally expensive training. Instead, the resources were used to fine-tune pretrained image classification models for the specific task. The evaluated models include Inception-v3,^40^ Inception-ResNet-v2,^41^ and EfficientNet-b0,^42^ because of their usability and compatibility with MATLAB integration on standalone workstations. The models were evaluated based on their ability to correctly classify EICs into the two groups, namely endogenous and background species, as well as the complexity of training and running the models, using quantitative measures of sensitivity and specificity. The sensitivity is defined as the ratio of the retained endogenous species (true positives) divided by the sum of the true positive species and lost endogenous species (false negatives). The specificity is the ratio of factual background removed (true negatives) divided by the sum of the true negatives and retained background (false positives). The labeling (annotation) of the training data required differentiation between the high S/N and low S/N classes, which is not defined by quantitative measures but is based solely on the visual EIC profile. Thus, the multiclass model was evaluated as a binary model using a confusion matrix, which compares the model’s predictions against the actual labels to show how often species were correctly or incorrectly classified (Figure S7). The performance metrics—accuracy, sensitivity, specificity, and training times—of the three prototype multiclass classifiers are summarized in Table 2 for validation on the unseen data set 4.

**Table 2:** Validation Results for Three Prototype DL Multiclass Classifiers

Model	Accuracy [%]	Sensitivity [%]	Specificity [%]	Training time [h]
Inception-v3	97	81	99	13.4
EfficicentNet-b0	97	89	98	4.6
Inception-ResNet-v2	98	88	98	42.0

The validation results show that all three models can achieve high prediction accuracies, while the required training time varies significantly. With a strong class imbalance toward the background species, accuracy becomes a less informative metric, making sensitivity—the retention of the underrepresented endogenous species—a more appropriate measure of performance. Specifically, the prototype models EfficientNet-b0 and Inception-ResNet-v2 achieved the highest sensitivities of 89% and 88%, respectively. This means that a high retention of endogenous species was achieved. In contrast to Inception-ResNet-v2, EfficientNet-b0 required only a fraction of the computation time to reach convergence, a stable state where the parameters (weights) barely change. Opposed to Inception-ResNet-v2 with 55.9 million parameters and 213 MB memory usage, EfficientNet-b0 uses only 5.3 million parameters and 20 MB memory. Introduced in 2019, the EfficientNet model series achieves state-of-the-art accuracy while significantly reducing the complexity, meaning the number of parameters, thereby enhancing network usability.^42^

To improve generalizability and performance on unseen data, EfficientNet-b0 was fine-tuned on a larger training set than the three previous prototype models. This training set combined features from three data sets (1, 2, and 3), each collected under different experimental conditions. To further expand and diversify the training and test data sets, the sequence and number of individual cells in the EIC plots of the limited endogenous species were systematically varied. Specifically, data sets were simulated with different magnification levels of EIC profiles: larger numbers of events produced sharper, centroid-like peaks, while smaller numbers generated broader, Gaussian-like peaks. For each species type, the number of events per EIC was adjusted from a minimum of 10 to a maximum of ∼100 in steps of 5, although profiles with as few as five events were also included to account for missing values. In total, this resulted in 3684 high S/N and 3952 low endogenous S/N species, as well as 4044 background training images. Following the model was validated on unseen data sets (4, 5, and 6) for a binary evaluation of the classification of endogenous features and background species (Table 3, Figure S8).

**Table 3:** Validation Results on Unseen Data for Different Conditions for the Binary Classification Evaluation of the Fine-Tuned EfficientNet-B0 Model

Condition	Accuracy [%]	Sensitivity [%]	Specificity [%]
Data set 4	97	95	98
Data set 5	96	96	97
Data set 6	99	98	99

The results show that the model assigned the correct characteristic with high accuracy, achieving at least 97% correct classifications across all three test data sets. Additionally, the model’s sensitivity was evaluated based on the number of retained endogenous analytes necessary to achieve high analyte coverage. A high sensitivity of 95% was achieved for the endogenous feature class. Thus, very few endogenous ions are lost by misclassification. The model’s specificity was evaluated based on the successful removal of background species from the endogenous feature class. The specificity shows that only 3% of the endogenous feature class was classified as background, showing a high-performing model. While no prior DL model exists for this kind of EIC classification, we note that the performance of this fine-tuned EfficientNet-b0 model is in line in terms of model performance with other fine-tuned EfficientNet-b0 models.^43,44^ For example, EfficientNet-b0 was fine-tuned to differentiate between malaria-parasitized and uninfected blood smears, achieving accuracies of up to 95%, with a sensitivity of 95% and a specificity of 94%.^43^ Overall, the DL model enables the SC platform to run at low filter thresholds during data processing, thereby achieving high coverage of analytes originating from single cells, including those of low abundance or frequency. The DL-based classification eliminates the need for manual inspection of all extracted species after data processing, thus generating trustworthy data with minimal operator time consumption.

### CellMate Operation

The entire workflow, from data import to data processing and DL-based classification, is collected in a GUI called CellMate. To maximize usability, CellMate enables easy and efficient operation with minimal user intervention and optimized run times. The GUI includes default settings, while still allowing the user to optimize the parameters based on their data set (Figure S3, S5, and S8). The results of the automatic cell identification (S3), the EIC of the extracted species (S5), and the DL classified species (S8) can be directly visually inspected in their respective tabs. Notably, the interactivity helps the user experience during cell identification (S3) or DL-based classification (S9). In both cases, incorrect classification or user preferences can be manually adjusted. The DL approach can also be applied to targeted (annotated/quantified) metabolite species to filter out *m*/*z* values populated by background species in the same mass channel as the targeted species (S10). This is particularly handy when dealing with a large database or searching for analytes of unknown presence. Thereby, all extracted features from the data processing tab (S5), regardless of the parameters, filter settings, or quantification/normalization used, can be loaded into the DL model for classification with their raw EIC profiles.

In addition to being user-friendly, CellMate also enables efficient processing of experiments with one hundred or more individual cells per cellogram, an important feature considering the way the data is acquired. Other than the increased number of sampled cells, which results in a longer acquisition time, the experiment conditions can also affect the complexity of the data. For example, adding dopants that increase detectability can vastly enhance the number of species that need to be processed. We assessed the computational time for the data processing and DL classification and the origin of the extracted species for the six data sets. The data processing was performed at default low filter thresholds, meaning an FC of 3 or 9 for very complex data sets (Data sets 2 and 6) and a DF of 33% for all six data sets. The results are shown in Figure S11, which displays the number of extracted *m*/*z* values, whether they are endogenous, and the total computation time, which is the sum of data processing and DL classification. The runtime of the process is primarily affected by the complexity of the data (Figure S11a), as a higher number of extracted *m*/*z* values will increase the runtime. In contrast to data processing, the increase in running time for DL classification is expected to be a linear response with the increasing number of features (0.2 s/species, R^2^ = 0.97, Figure S11a), as EIC plot generation and classification are performed sequentially. The data processing and DL classification can be completed in as little as 10 min for almost 1500 extracted *m*/*z* values, while processing over 3500 *m*/*z* values in under 30 min. This computational time is lower than previously reported by other SCM data processing platforms with seemingly similar data sets.^19^

Across the six processed data sets, the number of endogenous species varied between experiments. Additionally, the number of endogenous species was found to have a moderately strong correlation with the total number of detected species (R^2^ = 0.45, Pearson correlation coefficient (r) = 0.67) (Figure S12). The highest number of deisotoped endogenous species was 828, which were extracted from data set 4, where primarily lipids were targeted. In contrast, only 90 endogenous metabolites were extracted from data set 3. Thus, despite the positive correlation between the number of extracted features and endogenous features, the ratio of detected species and endogenous features can still vary across data sets. For filtering, this would require filter threshold optimization to efficiently filter background while retaining endogenous species in different data sets, which is not easily predictable. In contrast, the DL approach does not rely on filtering and operator-defined thresholds, which removes operator biases and increases time efficiency.

The comparison of the two approaches is visualized using the sensitivity and specificity with ideal values of 1. Specifically, the confusion of the actual and predicted classes of species was assessed with the complete pool of *m*/*z* values at low filter thresholds. The number of endogenous species was inferred using a low filter threshold of DF = 33% and an FC of 3, without any additional filtering steps, for both approaches. The results show that for the statistical filtering there is an inverse relationship between sensitivity and specificity, with low specificity at low thresholds, while retaining most endogenous species (Figure 5). However, achieving a high specificity at high filter thresholds (Figure 5b) results in a loss of more than 50% of endogenous species (Figure 5a), despite having very few background species. In contrast, the DL approach achieves a very high sensitivity (0.98) and specificity (0.98) simultaneously. Thus, the DL approach eliminates the need to adjust filter thresholds while retaining the precious endogenous species. Together, CellMate integrates interactive visualization, efficient data processing, and DL-based classification into a streamlined workflow that adapts to diverse experimental conditions. The GUI allows individual adjustments by the user while maintaining fast runtimes, even for large and complex data sets. Importantly, this enables the reliable identification of endogenous species across experiments, independent of their background complexity, thereby ensuring both the usability and robustness of the platform.

**Figure 5:**
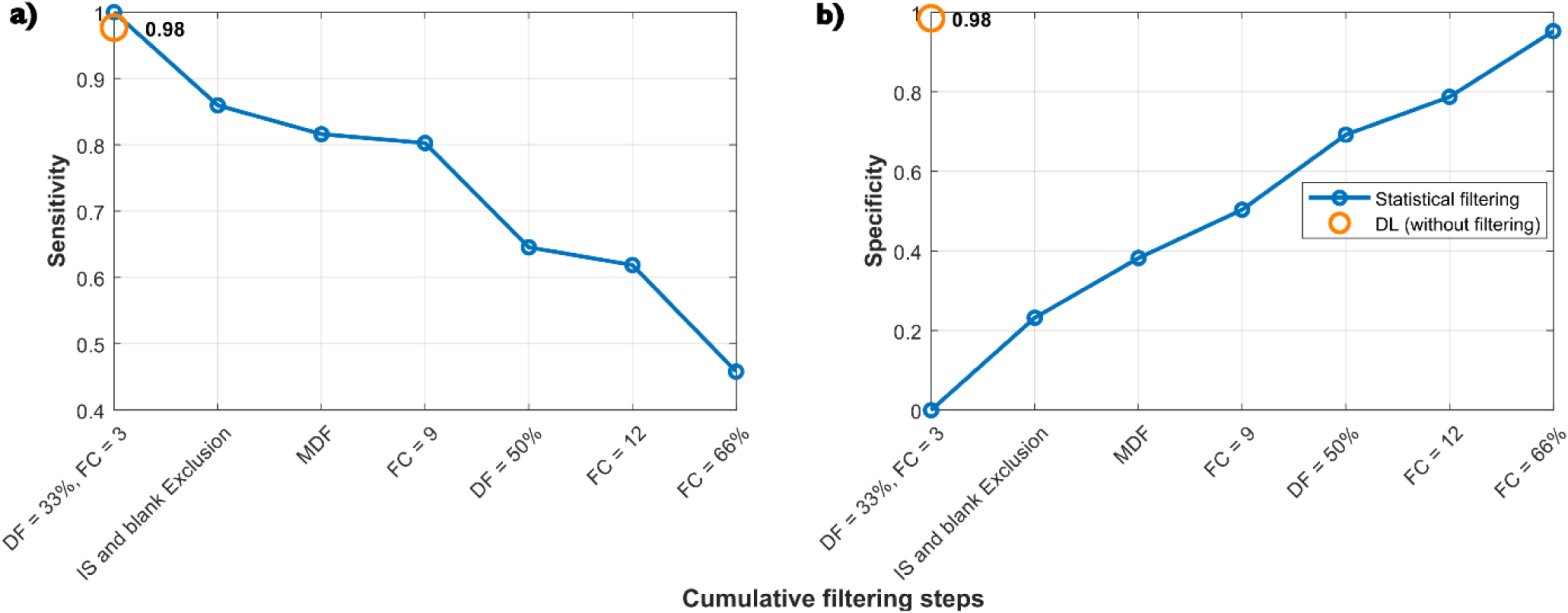
Comparison of the statistical filtering and the DL approach to eliminating background species. Statistical filtering is applied cumulatively in different steps and thresholds (blue). In contrast, the DL classification (orange) is applied at the lowest threshold settings (DF = 33%, FC = 3) without additional filtering steps during data processing. The ability to retain endogenous species of the two approaches is assessed as sensitivity (a), and the selective removal of background species is expressed as specificity (b).

## Conclusion

We presented the streamlined workflow embedded in the CellMate application, which provides easy and efficient access to complex direct infusion SCM data. Combining established processing routines with a novel DL classification algorithm paves the way for routine global metabolomics of single cells. Fast and comprehensive access is possible by using either ThermoFisher’s vendor-specific .RAW format or the universal .mzML format. The automated cellographic peak detection algorithm aids the data selection process and offers high customization. A wide variety of data processing options facilitates both targeted and untargeted approaches. Quantitative work is supported by pairing the respective *m*/*z* values of IS with their endogenous counterparts. Introducing a DL classifier in this application accelerates the unbiased exploration of the metabolome of interest. Finally, the interactive embedding of this workflow within the application enables adjustments and modifications. Thus, a new tool is available to address the overwhelming complexity of global metabolomics using DIMS. In particular, the SCM community will benefit from the high coverage of precious metabolite information from trace amounts while processing hundreds of individual cells swiftly.

## Supplementary Material



## Notes

Performance metrics, additional data, an overview of CellMate’s GUI, and workflow (PDF)
