# Supplementary material for: CellMate—A Deep Learning-Assisted Single-Cell Data Processing Platform
Source: Anal Chem. 2026 Feb 13;98(7):5561–9. doi: 10.1021/acs.analchem.5c07205 (PMC12937054; doi:10.1021/acs.analchem.5c07205)
Supplement: Supplementary file 1 [file ac5c07205_si_001.pdf]

Supporting information

CellMate – A Deep Learning-Assisted Single-Cell Data Processing Platform

Felix Friedrich[1], Cátia Marques[1], Ingela Lanekoff[1,2*]

[1] Department of Chemistry for Life Sciences, Uppsala University, Uppsala, Sweden
[2] Center of Excellence for the Chemical Mechanisms of Life, Uppsala University, Sweden

Corresponding author:
Prof. Ingela Lanekoff
Ingela.Lanekoff@kemi.uu.se
Department of Chemistry for Life Sciences
Uppsala University
75123 Uppsala
Sweden

Table of Contents

Figures 3

Tables 12

Figures

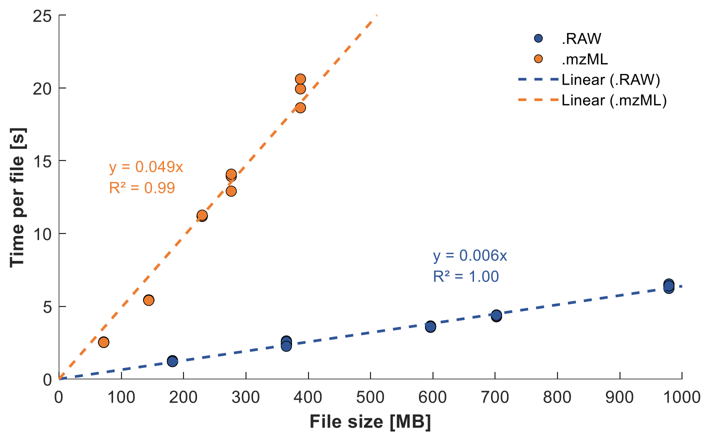

Figure S1. Comparison of the time needed to import data files for different file sizes via the .RAW (blue) and the .mzML format (orange). The time needed to read a file is plotted as a function of the file size for both import modalities. The .RAW files were imported via the RawFileReader .NET dynamic link libraries (ThermoFisher) while .mzML files were imported through an in-house developed function.[2] The .RAW import is about eight times faster (167 MB/s) than the .mzML import (20 MB/s).

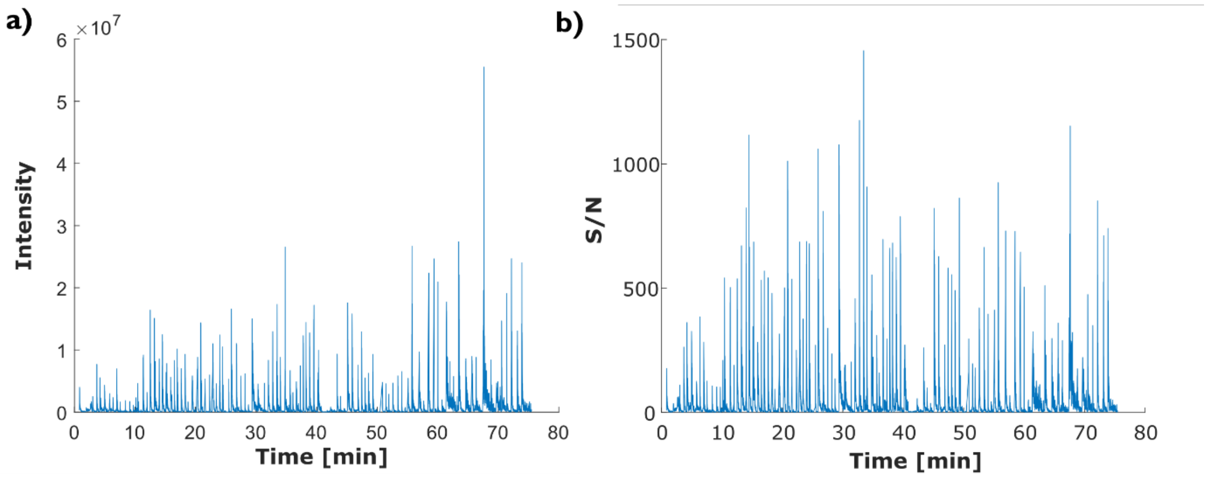

Figure S2. Comparison of two extracted ion chronograms of *m/z* 760.586 with plotted a) intensity and b) S/N ratio against the time for the pos. MeOH control data set. The S/N of each *m/z* species is defined as follows $S/N = \frac{Intensity - Baseline}{Noise - Baseline}$ with Intensity, Noise, and Baseline as the raw, noise, and baseline intensities of the *m/z* species in each scan extracted directly from the .RAW file.

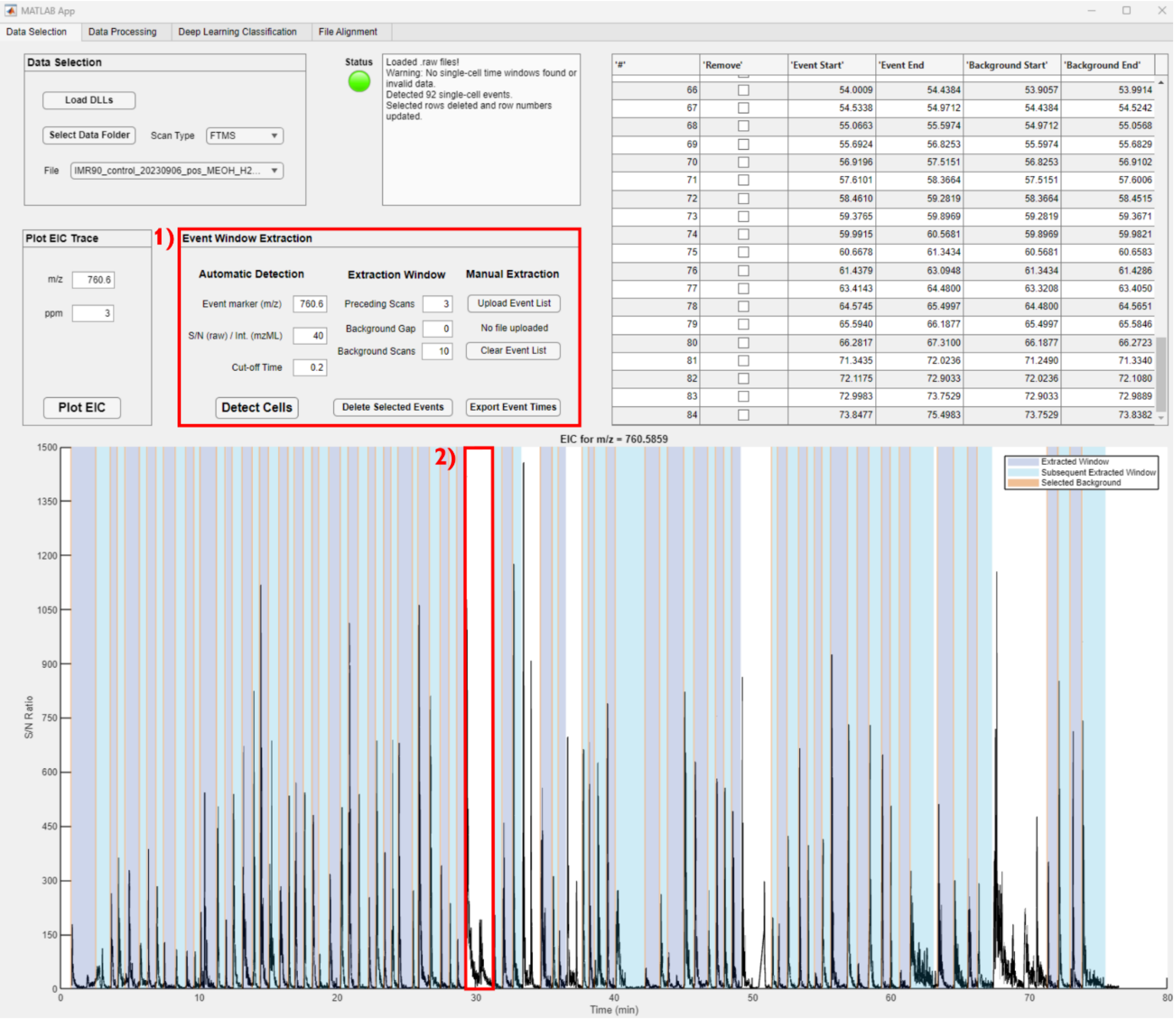

'#'	'Remove'	'Event Start'	'Event End'	'Background Start'	'Background End'
66	☐	54.0009	54.4384	53.9057	53.9914
67	☐	54.5338	54.9712	54.4384	54.5242
68	☐	55.0663	55.5974	54.9712	55.0568
69	☐	55.6924	56.8253	55.5974	55.6829
70	☐	56.9196	57.5151	56.8253	56.9102
71	☐	57.6101	58.3664	57.5151	57.6006
72	☐	58.4610	59.2819	58.3664	58.4515
73	☐	59.3765	59.8969	59.2819	59.3671
74	☐	59.9915	60.5681	59.8969	59.9821
75	☐	60.6678	61.3434	60.5681	60.6583
76	☐	61.4379	63.0948	61.3434	61.4286
77	☐	63.4143	64.4800	63.3208	63.4050
78	☐	64.5745	65.4997	64.4800	64.5651
79	☐	65.5940	66.1877	65.4997	65.5846
80	☐	66.2817	67.3100	66.1877	66.2723
81	☐	71.3435	72.0236	71.2490	71.3340
82	☐	72.1175	72.9033	72.0236	72.1080
83	☐	72.9983	73.7529	72.9033	72.9889
84	☐	73.8477	75.4983	73.7529	73.8382

Figure S3. GUI of the data selection tab and the highlighted automated event extraction box (1). The highlighted white background shows manually deleted extraction windows in which, for example, two cell measurements were merged into one cellogram (2).

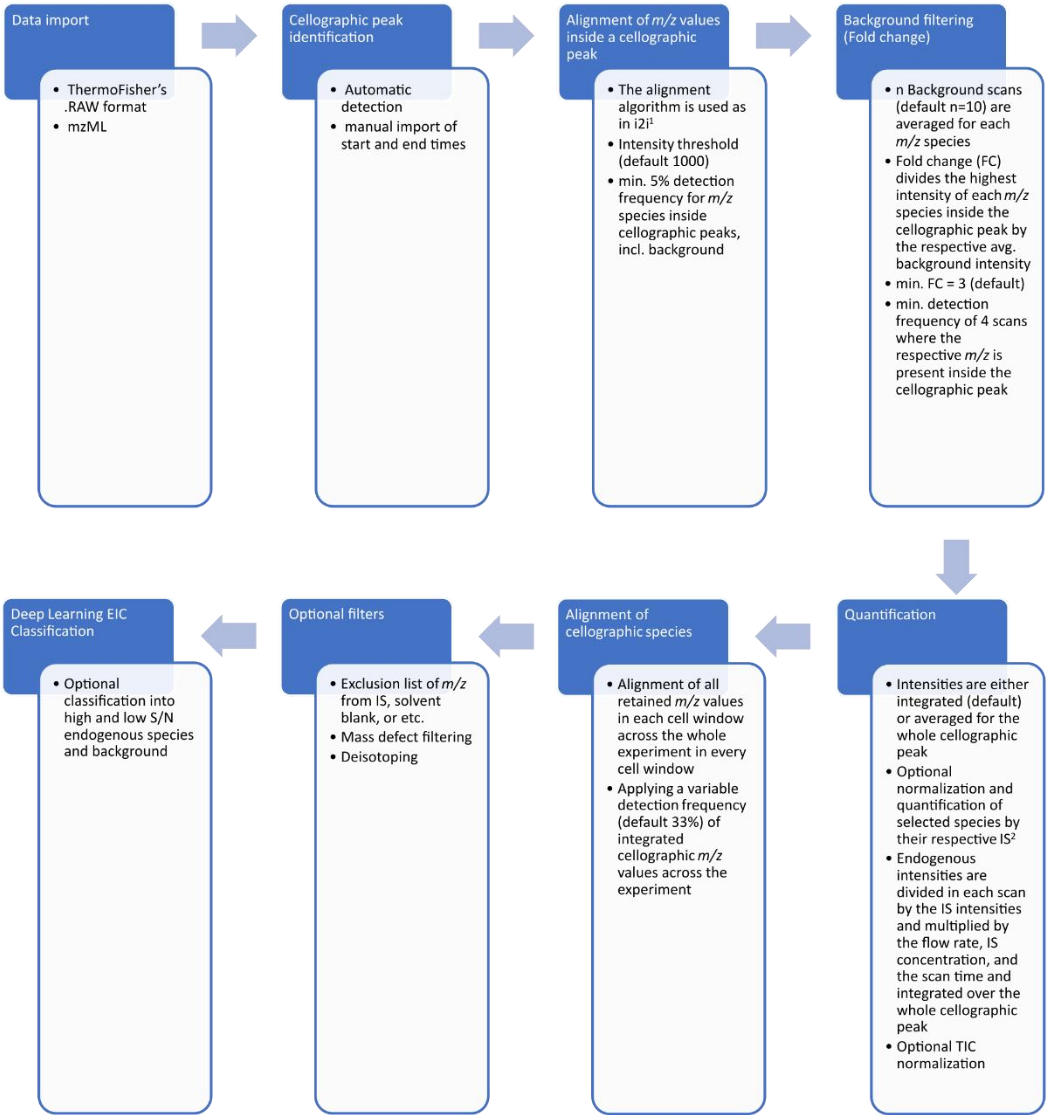

Figure S4. Flowchart of the CellMate workflow. All critical steps during the workflow are described from data import through data processing to DL classification. In many steps, default settings can be changed. Optional filters can be used for both targeted and non-targeted data processing.

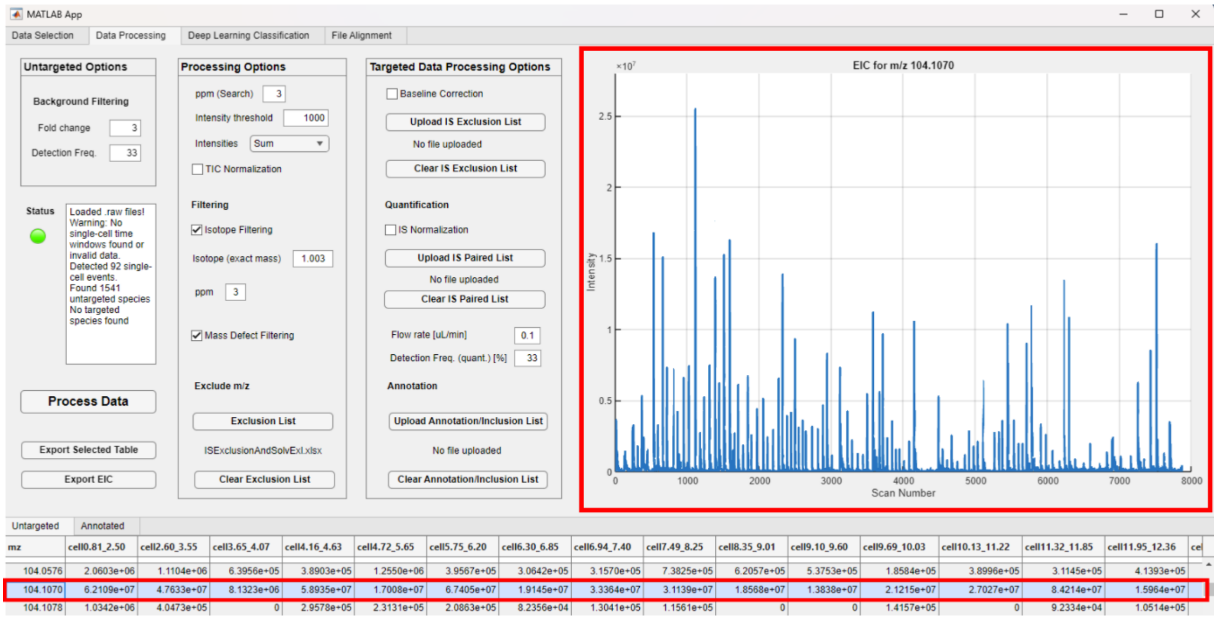

Figure S5. Data processing tab with EIC profiles of different extracted features. An extracted *m/z* value is selected in the table (red highlighted), and the EIC is plotted for the endogenous feature at *m/z* 104.107 (choline$^+$) as shown in the GUI.

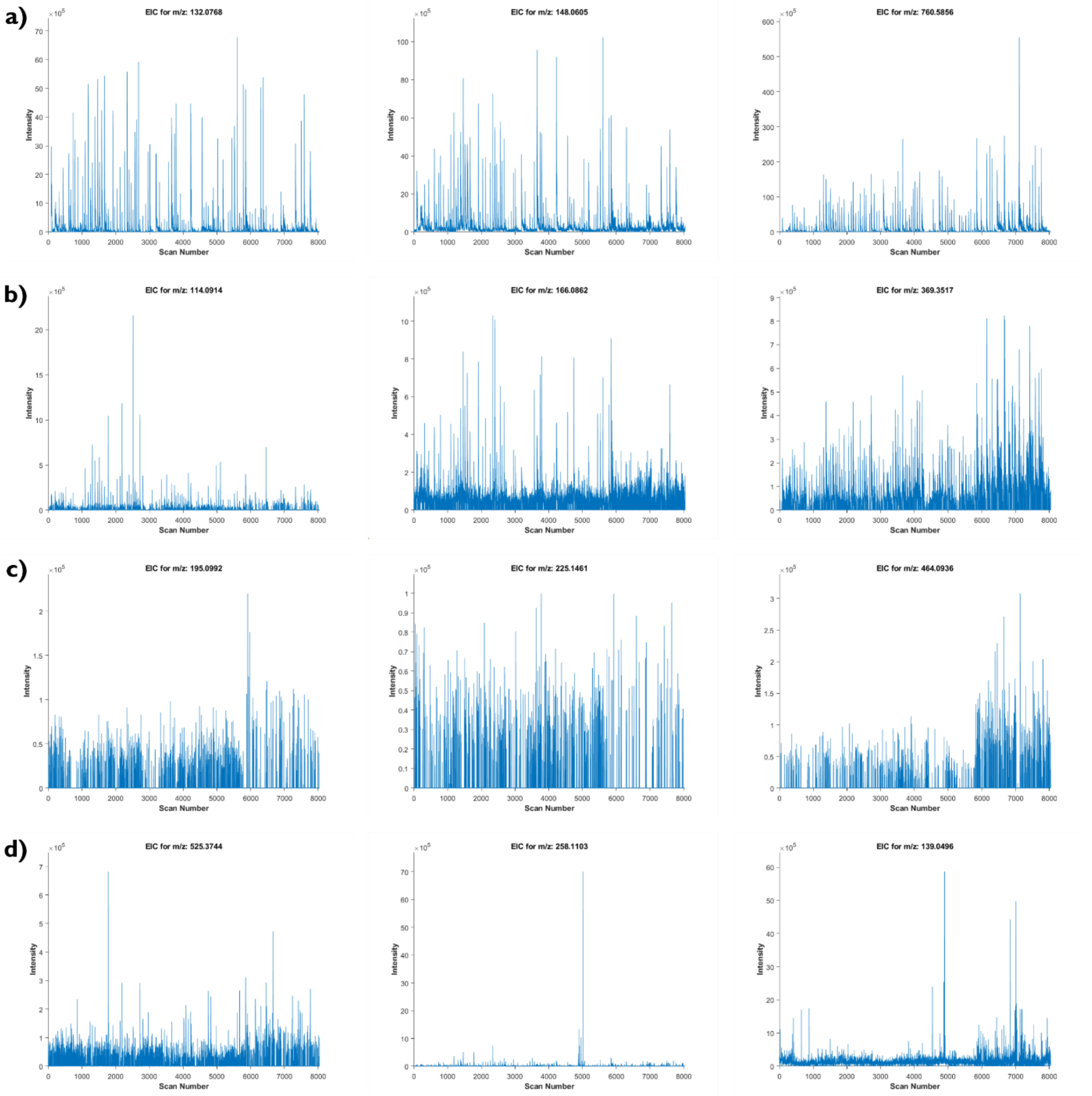

Figure S6. Multiple examples of EIC profiles of the multi-class classification for a) high S/N, b) low S/N, c) BG, and d) borderline cases of low S/N and BG species.

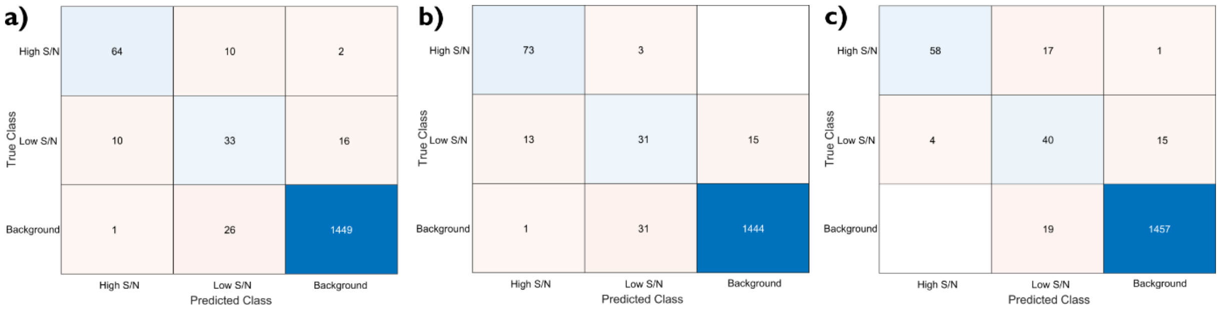

Figure S7. Confusion matrices for the multi-class classification of high S/N, low S/N, and BG species for three different prototyped fine-tuned models of a) Inception-v3, b) EfficientNet-b0, and c) Inception-ResNet-v2 on data set 4.

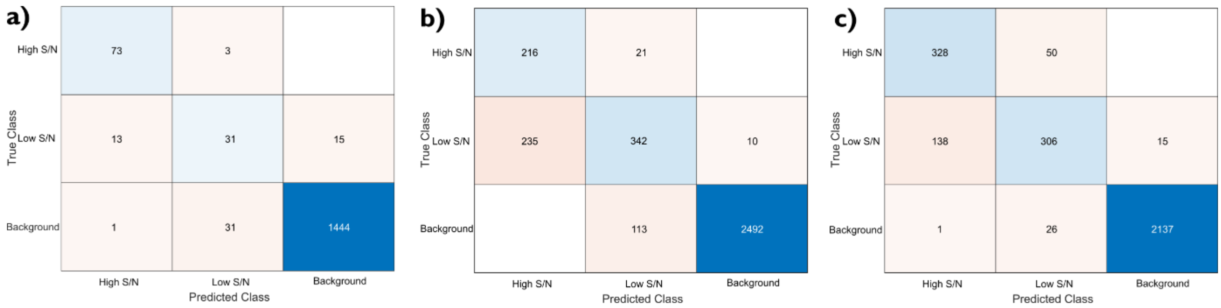

Figure S8. Confusion matrices for the multi-class classification of high S/N, low S/N, and BG species with the transfer-learned EfficientNet-b0 model for three different data sets of a) dataset 4, b) dataset 5, and c) dataset 6. The number of endogenous species was tripled for dataset 6 by shuffling and adjusting the number of single-cell events to approximately 15, 45, and 75 cells per EIC image.

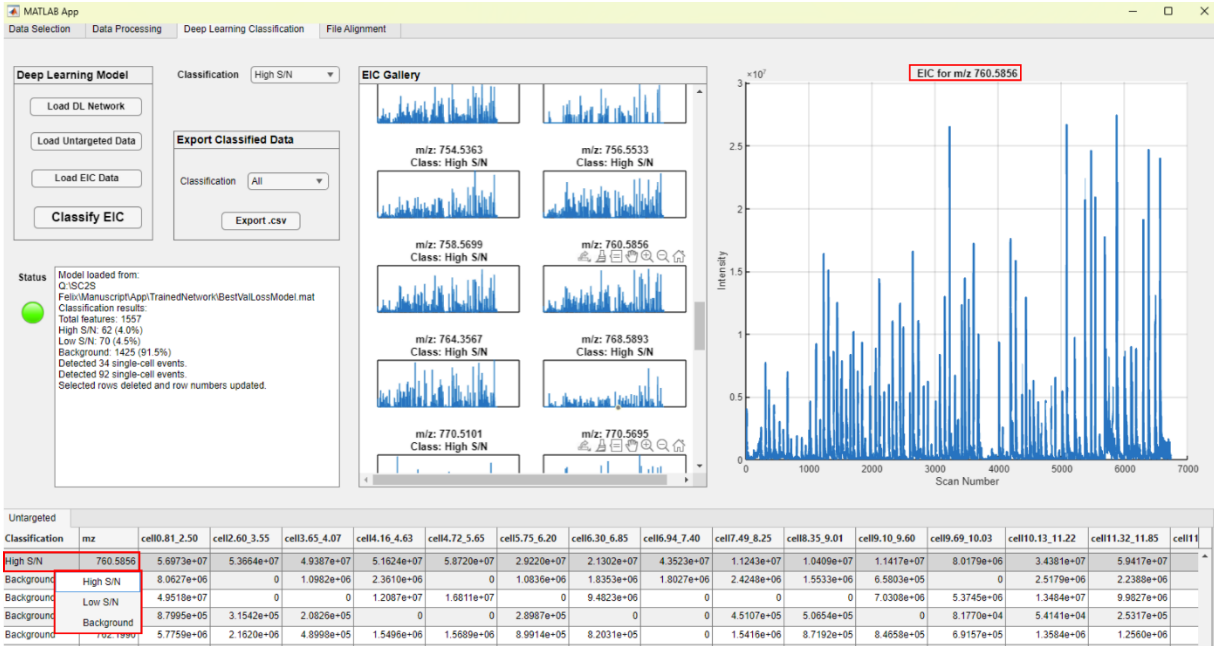

Figure S9. Deep-learning classification tab with classification and EIC profiles of different extracted *m/z* values. The extracted *m/z* values from the untargeted tab in Tab 2 are classified by a pre-trained DL model. The red-highlighted species at m/z 760.5859 is classified as a high S/N endogenous feature and is plotted with its corresponding EIC profile in high detail (right) and in the EIC gallery (left). Upon right-clicking, the classification can be changed to the user's preference in the displayed data table (bottom).

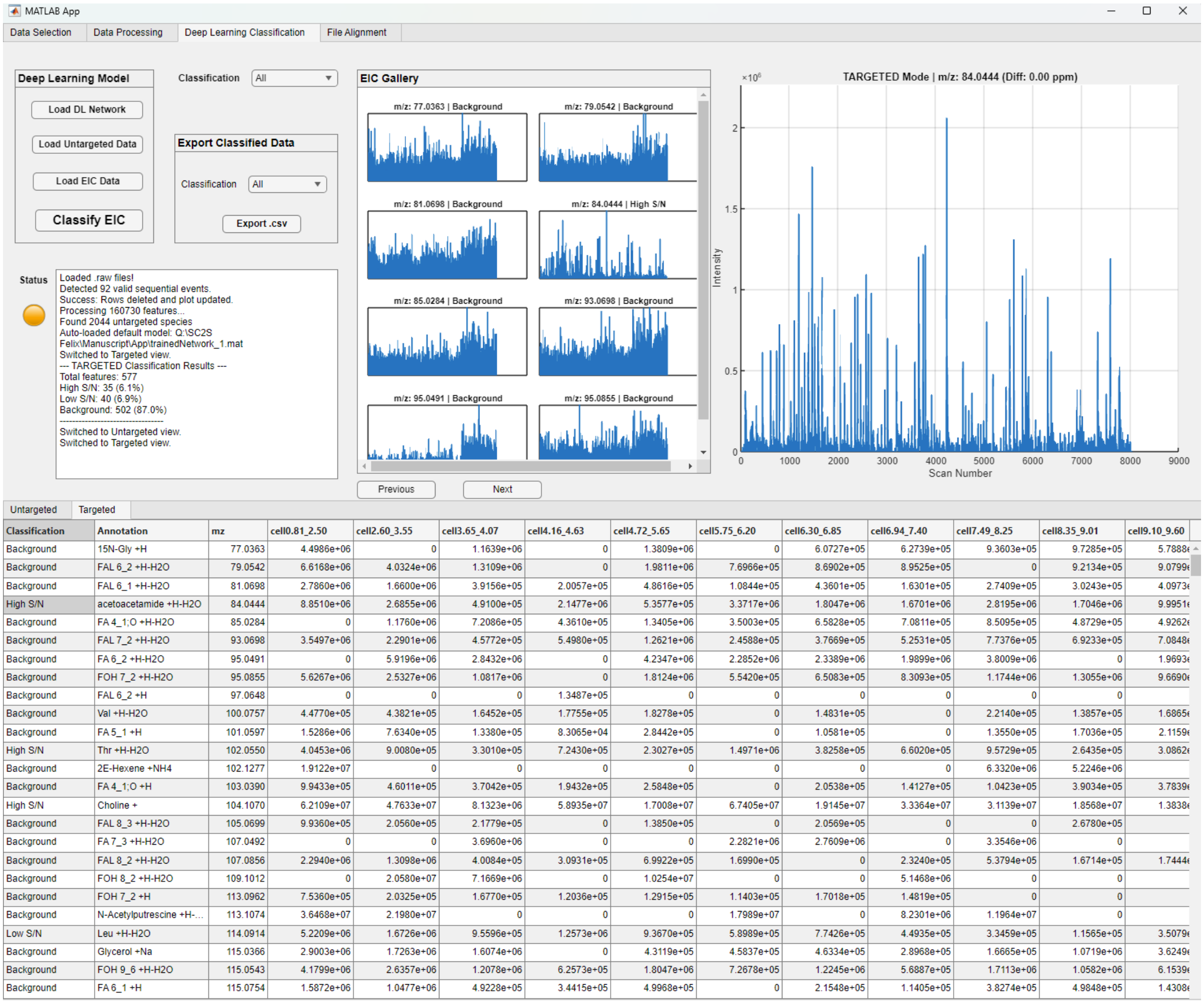

Classification	Annotation	mz	cell0.81_2.50	cell2.60_3.55	cell3.65_4.07	cell4.16_4.63	cell4.72_5.65	cell5.75_6.20	cell6.30_6.85	cell6.94_7.40	cell7.49_8.25	cell8.35_9.01	cell9.10_9.60
Background	15N-Gly +H	77.0363	4.4986e+06	0	1.1639e+06	0	1.3809e+06	0	6.0727e+05	6.2739e+05	9.3603e+05	9.7285e+05	5.7888e
Background	FAL 6_2 +H-H2O	79.0542	6.6168e+06	4.0324e+06	1.3109e+06	0	1.9811e+06	7.6966e+05	8.6902e+05	8.9525e+05	0	9.2134e+05	9.0799e
Background	FAL 6_1 +H-H2O	81.0698	2.7860e+06	1.6600e+06	3.9156e+05	2.0057e+05	4.8616e+05	1.0844e+05	4.3601e+05	1.6301e+05	2.7409e+05	3.0243e+05	4.0973e
High S/N	acetoacetamide +H-H2O	84.0444	8.8510e+06	2.6855e+06	4.9100e+05	2.1477e+06	5.3577e+05	3.3717e+06	1.8047e+06	1.6701e+06	2.8195e+06	1.7046e+06	9.9951e
Background	FA 4_1;O +H-H2O	85.0284	0	1.1760e+06	7.2086e+05	4.3610e+05	1.3405e+06	3.5003e+05	6.5828e+05	7.0811e+05	8.5095e+05	4.8729e+05	4.9262e
Background	FAL 7_2 +H-H2O	93.0698	3.5497e+06	2.2901e+06	4.5772e+05	5.4980e+05	1.2621e+06	2.4588e+05	3.7669e+05	5.2531e+05	7.7376e+05	6.9233e+05	7.0848e
Background	FA 6_2 +H-H2O	95.0491	0	5.9196e+06	2.8432e+06	0	4.2347e+06	2.2852e+06	2.3389e+06	1.9899e+06	3.8009e+06	0	1.9693e
Background	FOH 7_2 +H-H2O	95.0855	5.6267e+06	2.5327e+06	1.0817e+06	0	1.8124e+06	5.5420e+05	6.5083e+05	8.3093e+05	1.1744e+06	1.3055e+06	9.6690e
Background	FAL 6_2 +H	97.0648	0	0	0	1.3487e+05	0	0	0	0	0	0	
Background	Val +H-H2O	100.0757	4.4770e+05	4.3821e+05	1.6452e+05	1.7755e+05	1.8278e+05	0	1.4831e+05	0	2.2140e+05	1.3857e+05	1.6865e
Background	FA 5_1 +H	101.0597	1.5286e+06	7.6340e+05	1.3380e+05	8.3065e+04	2.8442e+05	0	1.0581e+05	0	1.3550e+05	1.7036e+05	2.1159e
High S/N	Thr +H-H2O	102.0550	4.0453e+06	9.0080e+05	3.3010e+05	7.2430e+05	2.3027e+05	1.4971e+06	3.8258e+05	6.6020e+05	9.5729e+05	2.6435e+05	3.0862e
Background	2E-Hexene +NH4	102.1277	1.9122e+07	0	0	0	0	0	0	0	6.3320e+06	5.2246e+06	
Background	FA 4_1;O +H	103.0390	9.9433e+05	4.6011e+05	3.7042e+05	1.9432e+05	2.5848e+05	0	2.0538e+05	1.4127e+05	1.0423e+05	3.9034e+05	3.7839e
High S/N	Choline +	104.1070	6.2109e+07	4.7633e+07	8.1323e+06	5.8935e+07	1.7008e+07	6.7405e+07	1.9145e+07	3.3364e+07	3.1139e+07	1.8568e+07	1.3838e
Background	FAL 8_3 +H-H2O	105.0699	9.9360e+05	2.0560e+05	2.1779e+05	0	1.3850e+05	0	2.0569e+05	0	0	2.6780e+05	
Background	FA 7_3 +H-H2O	107.0492	0	0	3.6960e+06	0	0	2.2821e+06	2.7609e+06	0	3.3546e+06	0	
Background	FAL 8_2 +H-H2O	107.0856	2.2940e+06	1.3098e+06	4.0084e+05	3.0931e+05	6.9922e+05	1.6990e+05	0	2.3240e+05	5.3794e+05	1.6714e+05	1.7444e
Background	FOH 8_2 +H-H2O	109.1012	0	2.0580e+07	7.1669e+06	0	1.0254e+07	0	0	5.1468e+06	0	0	
Background	FOH 7_2 +H	113.0962	7.5360e+05	2.0325e+05	1.6770e+05	1.2036e+05	1.2915e+05	1.1403e+05	1.7018e+05	1.4819e+05	0	0	
Background	N-Acetylputrescine +H-...	113.1074	3.6468e+07	2.1980e+07	0	0	0	1.7989e+07	0	8.2301e+06	1.1964e+07	0	
Low S/N	Leu +H-H2O	114.0914	5.2209e+06	1.6726e+06	9.5596e+05	1.2573e+06	9.3670e+05	5.8989e+05	7.7426e+05	4.4935e+05	3.3459e+05	1.1565e+05	3.5079e
Background	Glycerol +Na	115.0366	2.9003e+06	1.7263e+06	1.6074e+06	0	4.3119e+05	4.5837e+05	4.6334e+05	2.8968e+05	1.6665e+05	1.0719e+06	3.6249e
Background	FOH 9_6 +H-H2O	115.0543	4.1799e+06	2.6357e+06	1.2078e+06	6.2573e+05	1.8047e+06	7.2678e+05	1.2245e+06	5.6887e+05	1.7113e+06	1.0582e+06	6.1539e
Background	FA 6_1 +H	115.0754	1.5872e+06	1.0477e+06	4.9228e+05	3.4415e+05	4.9968e+05	0	2.1548e+05	1.1405e+05	3.8274e+05	4.9848e+05	1.4308e

Figure S10. Deep-learning classification tab with classification and EIC profiles of targeted (annotated) extracted *m/z* values. Both untargeted and targeted analytes can be classified based on the selected tab.

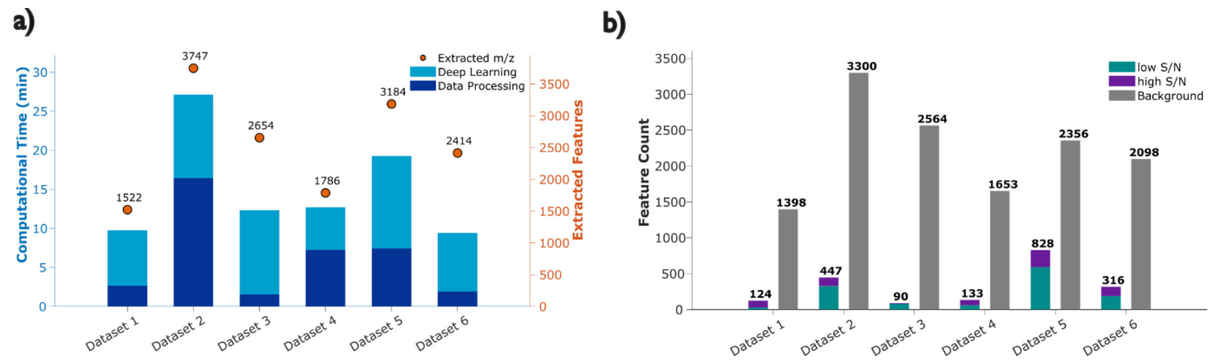

Figure S11. Evaluation of the running time of the data processing and DL-based classification, and the number and origin of the extracted *m/z* values at default settings. a) The computation time needed for the data processing (dark blue) and the deep learning classification time (light blue) for six different data sets with different complexity (experimental conditions, cell type, and number of extracted *m/z* values). c) The number of putative endogenous species with high S/N (purple), low S/N (teal), and background features (grey) for the six different data sets.

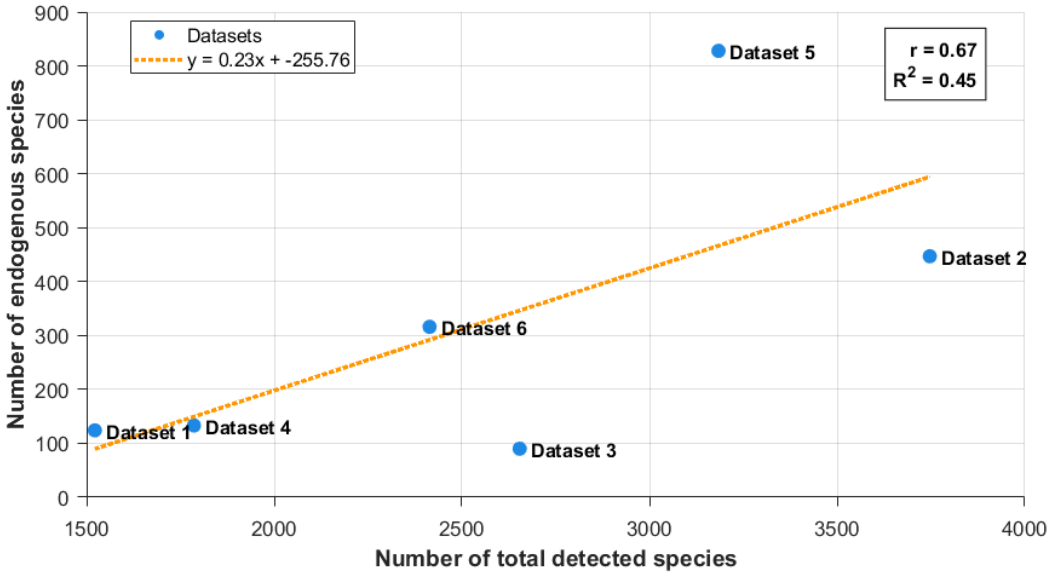

Figure S12. Ratio of the number of endogenous species and the number of total detected species for six datasets. The linear regression (orange) shows a moderately strong positive correlation (r = 0.67) between the number of endogenous species and the total number of detected species at low filter thresholds.

Tables

Table S1. Concentrations (µM) of standard solutions used in the MeOH:H_2O extraction solvent.

Internal standard	Concentration (µM)
LPC 19:0	4.7
PC 11:0/11:0	4.7
Acetylcholine-d_9	2.4
Glucose-d_2	8.3
GABA-d_2	5.6
Arachidonic-d_8	0.7
FA 18:1-d_9	4.3
Glutamate-d_3	0.7
MG 19:2	4.7
DG 26:0	4.3
carnitine-d_3	2.3
C18-carnitine-d_3	2.3
Alanine-^{15}N	5.9
Arginine-^{15}N	2.6
Asparagine-^{15}N	14.6
Aspartic acid-^{15}N	14.6
Cysteine-^{15}N	8.4
Glutamic acid-^{15}N	8.4
Glutamine-^{15}N	8.4
Glycine-^{15}N	10.1
Histidine-^{15}N	4.3
Isoleucine-^{15}N	4.9
Leucine-^{15}N	7.8
Lysine-^{15}N	3.0
Methionine-^{15}N	1.4
Phenylalanine-^{15}N	8.4
Proline-^{15}N	8.4
Serine-^{15}N	5.0
Threonine-^{15}N	5.7
Tryptophan-^{15}N	8.4
Tyrosine-^{15}N	1.7
Valine-^{15}N	6.3

Table S2. Concentration (µM) of standard solution used in the ACN:IPA extraction solvent.

Internal standard	Concentration (µM)
Pregnenolone-$^{13}C_2$, d_2	5
